# Oxidative deamination of lysine residues by polyphenols generates an equilibrium of aldehyde and 2-piperidinol products

**DOI:** 10.1016/j.jbc.2021.101035

**Published:** 2021-07-31

**Authors:** Kosuke Yamaguchi, Masanori Itakura, Roma Kitazawa, Sei-Young Lim, Koji Nagata, Takahiro Shibata, Mitsugu Akagawa, Koji Uchida

**Affiliations:** 1 Graduate School of Agricultural and Life Sciences, The University of Tokyo, Tokyo, Japan; 2 Graduate School of Bioagricultural Sciences, Nagoya University, Nagoya, Japan; 3 Graduate School of Life and Environmental Sciences, Osaka Prefecture University, Sakai, Japan; 4 Japan Agency for Medical Research and Development, CREST, Tokyo, Japan

**Keywords:** lysyl oxidation, polyphenol, post-translational modification, innate immunity, oxidation–reduction (redox), oxidative deamination of lysine, innate epitope, AAS, α-aminoadipic-5-semialdehyde, Bt-APA, *N*-biotinyl-5-aminopentylamine, D_2_O, heavy water, DMSO, dimethyl sulfoxide, EGCG, (-)-epigallocatechin-3-*O*-gallate, ESI, electrospray ionization, ESM, eggshell membrane, HRP, horseradish peroxidase, IgM, immunoglobulin M, mAb, monoclonal antibody, NaBH4, sodium borohydride, PBST, PBS containing 0.05% Tween-20, PQQ, pyrroloquinoline quinine, TFA, trifluoroacetic acid, UPLC, ultraperformance LC

## Abstract

Polyphenols, especially catechol-type polyphenols, exhibit lysyl oxidase–like activity and mediate oxidative deamination of lysine residues in proteins. Previous studies have shown that polyphenol-mediated oxidative deamination of lysine residues can be associated with altered electrical properties of proteins and increased crossreactivity with natural immunoglobulin M antibodies. This interaction suggested that oxidized proteins could act as innate antigens and elicit an innate immune response. However, the structural basis for oxidatively deaminated lysine residues remains unclear. In the present study, to establish the chemistry of lysine oxidation, we characterized oxidation products obtained *via* incubation of the lysine analog *N*-biotinyl-5-aminopentylamine with eggshell membranes containing lysyl oxidase and identified a unique six-membered ring 2-piperidinol derivative equilibrated with a ring-open product (aldehyde) as the major product. By monitoring these aldehyde–2-piperidinol products, we evaluated the lysyl oxidase–like activity of polyphenols. We also observed that this reaction was mediated by some polyphenols, especially *o*-diphenolic-type polyphenols, in the presence of copper ions. Interestingly, the natural immunoglobulin M monoclonal antibody recognized these aldehyde–2-piperidinol products as an innate epitope. These findings establish the existence of a dynamic equilibrium of oxidized lysine and provide important insights into the chemopreventive function of dietary polyphenols for chronic diseases.

Oxidative modification of proteins is associated with age-related pathologies, such as atherosclerosis, neurodegenerative disorders, and cataracts (1, 2). Such modifications can be mediated by nonenzymatic systems, including metal-catalyzed oxidation of reducing agents. The reaction process involves the reduction of metal ions with the appropriate electron donor (reducing agent) and the production of reactive oxygen species that lead to the oxidation of amino acid residue side chains. The ε-amino group of lysine is a potential target for oxidative modification and is known to form oxidation products, such as α-aminoadipic-5-semialdehyde (AAS), with carbonyl functionality (Fig. 1*A*) (3, 4). Lysyl oxidase, a copper-dependent amine oxidase, is also known to catalyze the oxidative deamination of lysine residues to generate AAS and plays an important role in the biogenesis of connective tissue matrices by crosslinking the extracellular matrix proteins, such as collagen (5, 6). Other enzyme-coupled mechanisms, including peroxidase, catechol, and hydrogen peroxide mixtures, have also been shown to convert lysine residues to AAS in proteins (7, 8, 9).

**Figure 1:**
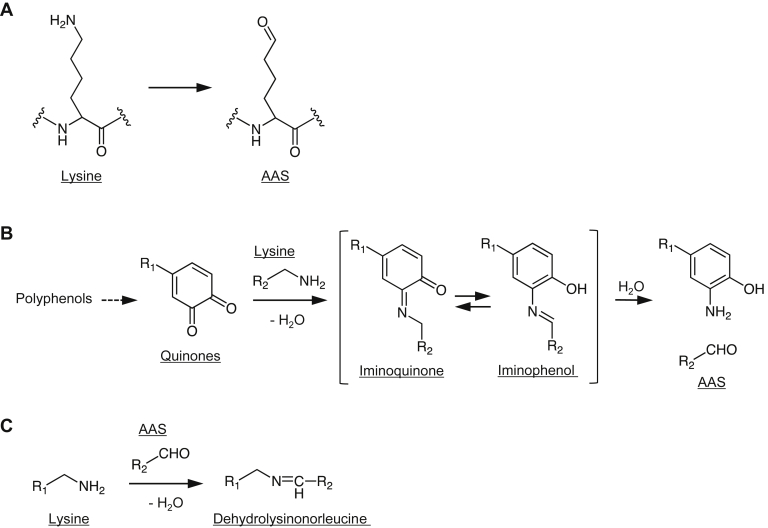
**Oxidative deamination of lysine residues.***A*, conversion of lysine to AAS. *B*, details of the mechanism of oxidative deamination of lysine. *o*-Quinones covalently bind to the lysine residues to form Schiff-base intermediates, such as iminoquinone and iminophenol, which are then converted to aminated polyphenols and deaminated product (AAS). *C*, formation of a lysine-derived crosslink dehydrolysinonorleucine. Crosslinking is formed by a Schiff-base reaction of AAS with the ε-amino group of unoxidized lysine residues. AAS, α-aminoadipic-5-semialdehyde.

Polyphenols, the most diverse group of phytochemicals, have received a great deal of attention as potential chemopreventive agents for chronic diseases, such as heart disease, cancer, diabetes, stroke, and arthritis. They are considered as antioxidants in general. However, some polyphenols, especially catechol-type polyphenols, acquire new functions by oxidation to the corresponding *o*-quinone derivatives that can catalyze oxidative deamination of primary amines (10). Indeed, the *o*-quinone derivative, pyrroloquinoline quinone (PQQ), is known to be an efficient mediator of the oxidation of primary amines such as ε-amino group of lysine residues in elastin and collagen (11, 12). Details of the mechanism of oxidative deamination have been previously proposed: *o*-quinones covalently bind to the lysine residues of proteins to form Schiff-base intermediates, such as iminoquinone and iminophenol, followed by the conversion to both aminated polyphenols and the deaminated product, AAS (Fig. 1*B*) (13, 14, 15). In our previous study using a click chemistry-based approach, we revealed the binding of (-)-epigallocatechin-3-*O*-gallate (EGCG), the most abundant polyphenol in green tea, to proteins, where EGCG oxidizes specific lysine residues in the protein (16). In addition, we studied the polymerization of proteins mediated by piceatannol, a naturally occurring hydroxylated analog of a red wine polyphenol resveratrol, and found that the polymerization was associated with the formation of a lysine-derived crosslink, dehydrolysinonorleucine (Fig. 1*C*) (17). More interestingly, we have shown that polyphenols, including EGCG, can be a source of innate epitopes recognized by natural immunoglobulin M (IgM) antibodies and suggested a possible mechanism by which the electronegativity of polyphenol-treated proteins might be involved in antibody recognition (16, 17). Despite these unique properties of polyphenol-treated proteins, the details of the underlying molecular mechanism for oxidative deamination by polyphenols remain unclear.

In the present study, to establish the lysine oxidation chemistry, a lysine analog, *N*-biotinyl-5-aminopentylamine (Bt-APA), was used as the substrate for oxidation by eggshell membranes (ESMs) containing lysyl oxidase (18, 19). We used this biotin-linked model to facilitate characterization of identification of innate oxidation-specific epitopes by ELISA. First, we studied how lysyl oxidase oxidizes the biotin-linked model and unequivocally established the existence of a unique 6-membered ring 2-piperidinol product as an equilibrium form of the aldehyde intermediate. We then investigated whether aldehyde–2-piperidinol products could be formed by polyphenols in the presence and absence of copper ions. Of interest, these products were found to function as innate antigens recognized by the natural IgM monoclonal antibody (mAb). These findings suggest that there is a dynamic equilibrium of oxidatively deaminated lysine between the ring-open and ring-closed intermediates in the polyphenol-oxidized proteins. They may also provide important insights into the involvement of polyphenols with oxidative deamination activity that trigger the innate immune response.

## Results

### Lysyl oxidase–mediated oxidation of Bt-APA

**Figure 2:**
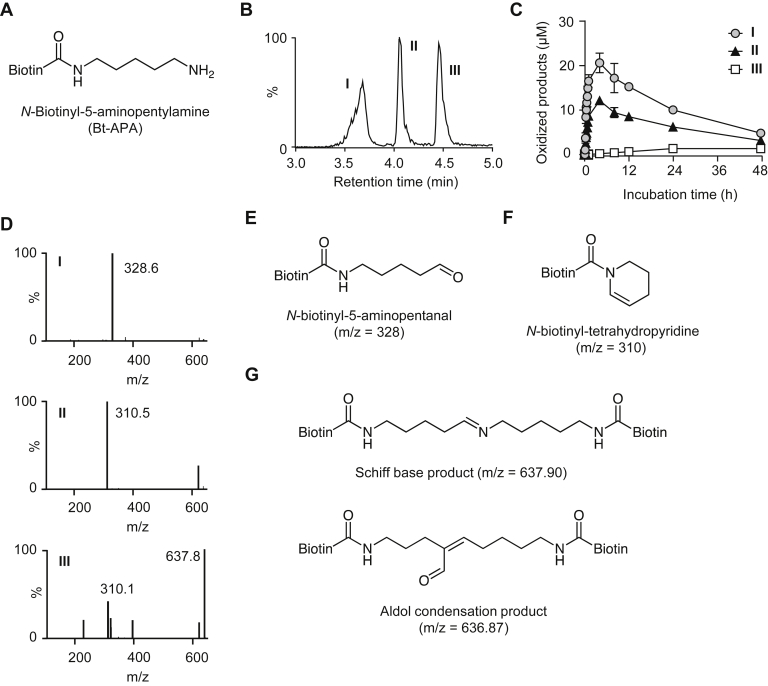
**Oxidation of Bt-ABA by lysyl oxidase.***A*, chemical structure of Bt-APA. *B*, LC–MS total ion chromatogram of oxidation products. Bt-APA (3 mM) was incubated with the ESM (10 mg/ml) in 0.1 M sodium phosphate buffer (pH 7.4) for 48 h at 37 °C. The products were monitored by LC–MS in the positive ion mode. *C*, time-dependent formation of products I to III. The yields of the products were calculated by comparing the peak area of the products with the initial area of the nonoxidized Bt-APA. *D*, mass spectra of products I to III. *E*, predicted structures of product I. *F*, the first predicted structure for product II. *G*, two possible structures of product III. Bt-APA, *N*-biotinyl-5-aminopentylamine; ESM, eggshell membrane.

To gain structural insights into the polyphenol-mediated oxidation of lysine residues, the lysine analog (Bt-APA) (Fig. 2*A*) was incubated with hen's ESM containing lysyl oxidase in 0.1 M sodium phosphate buffer for 48 h, and the reaction mixtures were analyzed by LC–MS. As shown in Figure 2, *B* and *C*, incubation with ESM gave three supposedly oxidized Bt-APAs; two major products (I and II) and one minor product (III). Incubation of the lysine analog with polyphenols and *o*-quinone compounds, such as PQQ, also generated the same products (data not shown).

Molecular-ion MS information about the products was collected using LC–MS. The mass spectrum of I showed an M + H^+^ at *m*/*z* 328 (Fig. 2*D*, *top panel*), suggesting that the product might be identical to the oxidatively deaminated aldehyde species, *N*-biotinyl-5-aminopentanal (Fig. 2*E*). This predicted structure was confirmed by the detection of the signal (9.52 ppm), corresponding to the aldehyde group, in the NMR spectra. The other major product II gave an intense M + H^+^ at *m*/*z* 310 on LC–MS analysis (Fig. 2*D*, *middle panel*), suggesting further dehydration of product I. Based on MS data, it was initially predicted that II might have a tetrahydropyridine structure (Fig. 2*F*). Kijewska *et al.* (20) recently studied the dehydrated form of aldehyde species and showed that peptides comprising aldehyde intermediates undergo side reactions to form tetrahydropyridine products. However, as far as we know, no such product with a tetrahydropyridine structure has been detected in lysyl oxidase–mediated oxidation of lysine residues. Therefore, at this point, the structure of product II remained unknown. On the other hand, the mass spectrum of III exhibited an intense M + H^+^ at *m*/*z* 637.8 (Fig. 2*D*, *bottom panel*), corresponding to either a Schiff base–type (imine) linkage (*m*/*z* 638) between Bt-APA and aldehyde product or aldol condensation (*m*/*z* 637) of two aldehyde products (Fig. 2*G*). The product was finally identified as an aldol condensation product based on NMR detection at two proton signals (6.67 and 9.12 ppm), corresponding to the α,β-unsaturated aldehyde.

### Identification of product II

**Figure 3:**
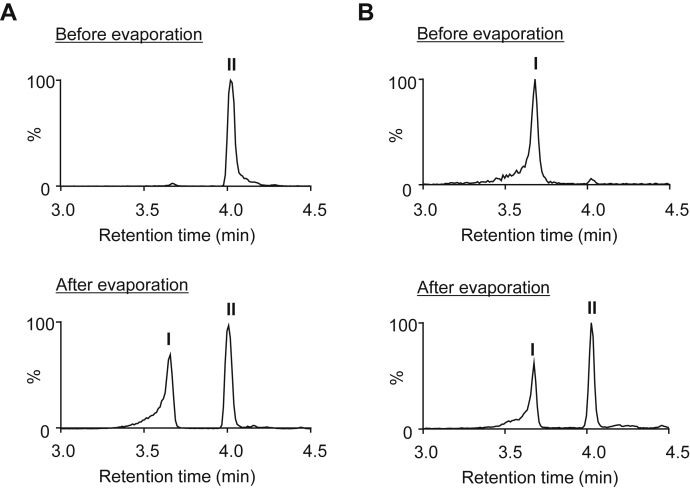
**Equilibrium relationship between I and II.***A*, the effect of evaporation on product II. After isolation by HPLC, the solution of product II was evaporated, dissolved with water, and then analyzed by LC–MS. Chromatograms (total ion): *upper*, before evaporation; *lower*, after evaporation. *B*, the effect of evaporation on product I. After isolation by HPLC, the solution of product I was evaporated, dissolved with water, and then analyzed by LC–ESI–MS. Chromatograms (total ion): *upper*, before evaporation; *lower*, after evaporation. ESI, electrospray ionization.

**Figure 4:**
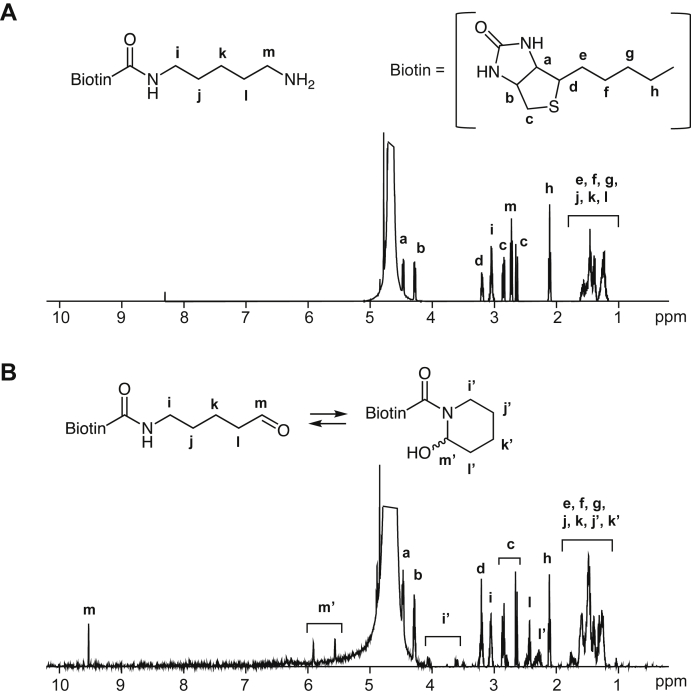
^**1**^**H-NMR analysis of a lysine analog and its oxidation products.***A*, ^1^H-NMR analysis of Bt-APA. *B*, ^1^H-NMR analysis of aldehyde (I)/piperidinol (II) products. Bt-APA, *N*-biotinyl-5-aminopentylamine.

**Figure 5:**
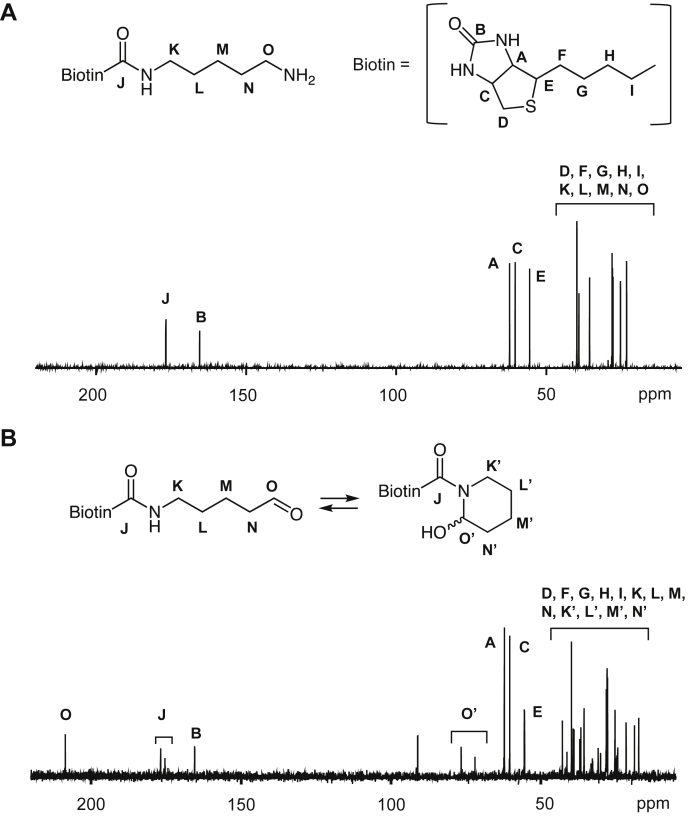
^**13**^**C-NMR analysis of a lysine analog and its oxidation products.***A*, C^13^-NMR analysis of Bt-APA. *B*, ^13^C-NMR analysis of aldehyde (I)/piperidinol (II) products. Bt-APA, *N*-biotinyl-5-aminopentylamine.

To elucidate product II, the lysine analog was treated with ESM and the product was isolated by HPLC on a reverse-phase column. After evaporation, however, the isolated sample (II) gave I in addition to II (Fig. 3*A*). Similarly, the evaporation of isolated I resulted in both I and II (Fig. 3*B*), suggesting that both products are in equilibrium in solution. The isolated sample that appeared to be a mixture of I and II was then characterized by NMR. Compared with the ^13^H-NMR spectrum between the original lysine analog and the sample (Fig. 4), a new signal at 9.52 ppm, corresponding to the aldehyde group, appeared in the ^1^H-NMR spectrum of the product. It was suggested that the signal might be originated from the aldehyde species (product I). Curiously, the characteristic signals of the olefinic protons and carbons of tetrahydropyridine were not detected in the NMR analysis of the product. Instead, ^1^H-NMR of the product detected two signals at 5.55 and 5.90 ppm and ^13^C-NMR detected two signals at 71.92 and 76.507 ppm (Fig. 5). These signals are commonly observed in the hemiacetal methine region. Therefore, it was speculated that II might not be a tetrahydropyridine product, but its undehydrated precursor, *N*-biotinyl-2-piperidinol. The detection of the two signals at the methine group in ^1^H-NMR and ^13^C-NMR analysis is consistent with the fact that the hemiaminal product is a mixture of diastereomers in solution. The discrepancy between the M + H^+^ signals of the product predictions (*m/z* = 328) and observations (*m/z* = 310) can be explained by the dehydration of the product in the positive ionization mode of electrospray ionization (ESI)–MS. In fact, the negative ionization mode of the ESI–MS showed an M − H^+^ signal at *m*/*z* 326 (data not shown).

**Figure 6:**
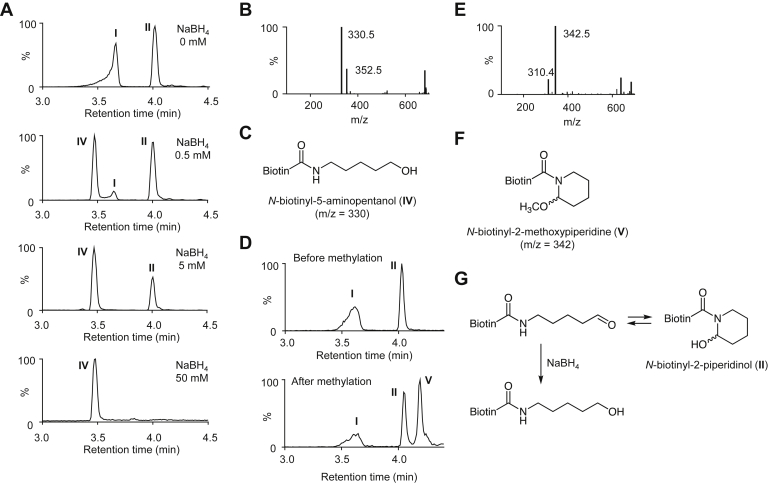
**Reversible equilibrium of products I and II.***A*, concentration-dependent reduction of the mixture containing aldehyde (I)/piperidinol (II) products with NaBH_4_. The mixture (10 μg/ml) of aldehyde (I)/piperidinol (II) products was incubated with NaBH_4_ (0–50 mM) in 0.1 M phosphate buffer (pH 7.4) for 2 h on ice. The reaction mixture was analyzed by LC–MS. Each profile displays the total ion chromatogram. *B*, mass spectrum of the reduced product IV. *C*, chemical structure of *N*-biotinyl-5-aminopentanol (IV). *D*, methylation of the mixture containing I and II. The mixture of aldehyde (I)/piperidinol (II) products was incubated in 1 ml methanol containing 0.1% trifluoroacetic acid for 12 h at room temperature. The reaction mixture was evaporated, dissolved with water, and then analyzed by LC–MS. Each profile displays the total ion chromatogram. *E*, mass spectrum of product V. *F*, chemical structure of *N*-biotinyl-2-methoxypiperidine. *G*, reduction of aldehyde–piperidinol products with NaBH_4_. NaBH_4_, sodium borohydride.

On the other hand, the hemiaminal ring opens and reforms, giving products with different configurations at the anomeric center. To identify the putative hemiaminal product, the isolated sample comprising ring-open and ring-closed structures was treated with sodium borohydride (NaBH_4_), and their reactivity for reduction reaction was examined. As shown in Figure 6*A*, the reduction profile was dependent on the concentration of NaBH_4_. When the sample was treated with the lowest concentration (0.5 mM) of the reducing agent, the product I was selectively lost along with the appearance of a new product at 3.47 min, which was determined to be a reduced form of the aldehyde species, *N*-biotinyl-5-aminopentanol (IV), by the MS analyses (Fig. 6, *B* and *C*). Treatment with 5 mM NaBH_4_ resulted in complete and partial loss of I and II, respectively, and only a new product (*N*-biotinyl-5-aminopentanol) was detected (Fig. 6*A*). At the highest concentration (50 mM) of NaBH_4_, both I and II were converted to *N*-biotinyl-5-aminopentanol (Fig. 6*A*). In addition, methylation of II with methanol/trifluoroacetic acid (TFA) gave a product (retention time of 4.20 min) with M + H^+^ at *m*/*z* 342 (Fig. 6, *D* and *E*), corresponding to *N*-biotinyl-2-methoxypiperidine (Fig. 6*F*). Based on all these data, the product equilibrated with the aldehydic species putatively presumed to be the tetrahydropyridine product (Fig. 2*F*) was finally determined to be the 2-piperidinol product (Fig. 6*G*).

### Detection of 2-piperidinol product in oxidized lysine residues

**Figure 7:**
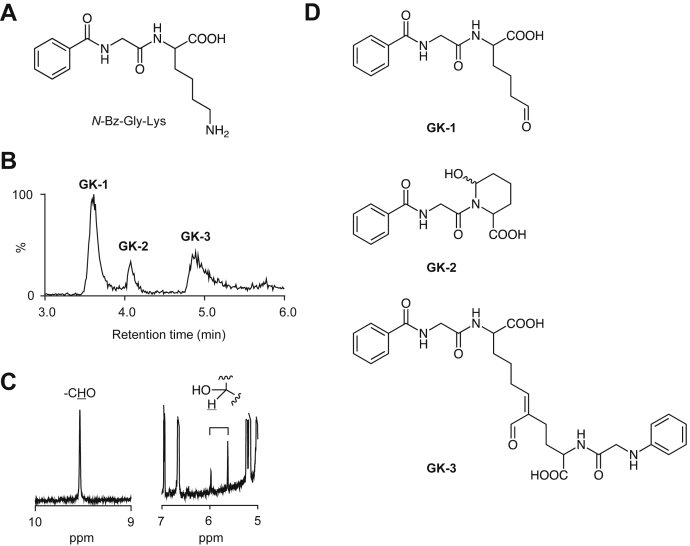
**Oxidation of a lysine-containing peptide by lysyl oxidase.***A*, chemical structure of *N*^α^-benzoylglycyl-lysine. *B*, LC–MS total ion chromatogram of oxidation products. *N*^α^-benzoylglycyl-lysine (3 mM) was incubated with the ESM (10 mg/ml) in 0.1 M sodium phosphate buffer (pH 7.4) for 24 h at 37 °C. The products were monitored by LC–MS in the positive ion mode. *C*, portions of ^1^H-NMR spectrum in the mixture of the putative aldehyde and 2-piperidinol products. Characteristic signals of the aldehyde (*left*) and hemiacetal methine (*right*) are shown. *D*, chemical structures of products GK-1, GK-2, and GK-3. ESM, eggshell membrane.

To demonstrate that the 2-piperidinol structure is generally formed by oxidative modification of lysine residues, a lysine-containing dipeptide, *N*^α^-benzoylglycyl-lysine, was incubated with ESM in 0.1 M sodium phosphate buffer for 48 h, and the products were analyzed by LC–MS. Three major products, GK-1, GK-2, and GK-3, with M + H^+^ at *m*/*z* 307, 434, and 596, respectively, were detected. They were speculated to be the products of aldehyde (GK-1), 2-piperidinol (GK-2), and aldol condensation (GK-3). When a mixture of the putative aldehyde and the 2-piperidinol product separated by HPLC was subjected to ^1^H-NMR, the characteristic signals of the aldehyde (9.53 ppm) and hemiacetal methine region (5.55 and 5.90 ppm) were indeed detected in the oxidized peptide (Fig. 7). Therefore, the equilibrium between ring-open and ring-closed forms could occur at the oxidized lysine residues of peptides.

**Figure 8:**
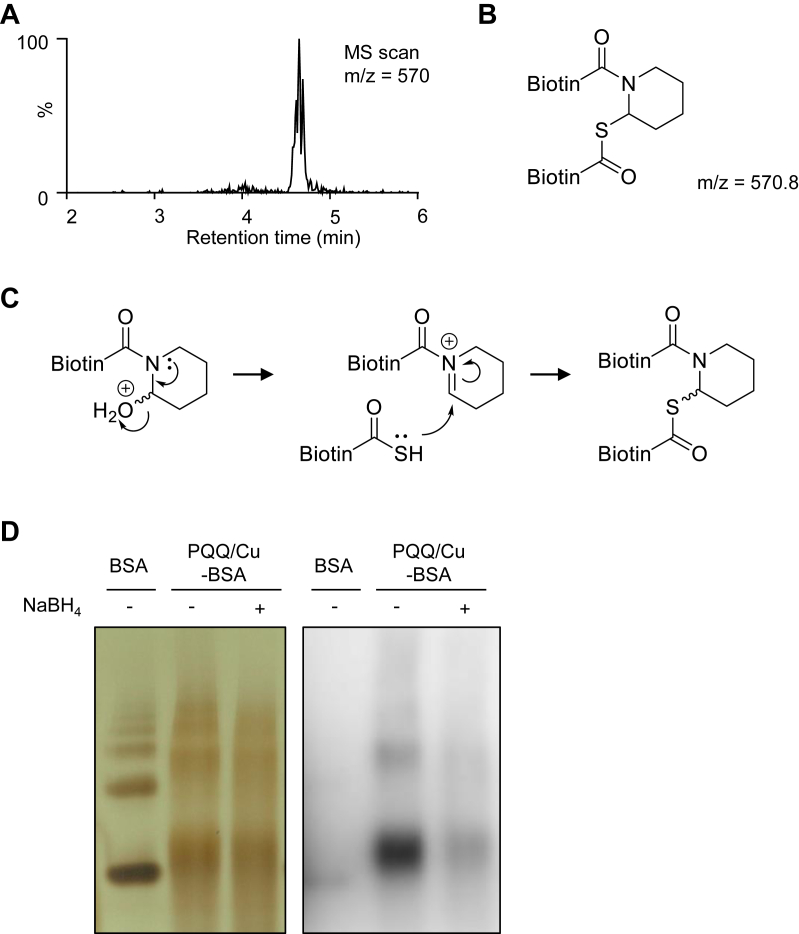
**Detection of 2-piperidinol product in oxidized proteins.***A*, selected ion current chromatogram obtained from LC–MS analysis of 2-piperidine thioester derivatives monitored with *m/z* 570. 2-Piperidinol (II) (50 μM) was treated with 2.5 mM biotin thioacid in 50 mM Na_2_CO_3_ for 2 h on ice, and the reaction mixture was subjected to LC–MS analysis. *B*, chemical structure of a 2-piperidine thioester derivative. *C*, mechanism of formation of 2-piperidine thioester derivative generated upon the reaction of 2-piperidinol (II) with biotin thioacid. *D*, detection of 2-piperidinol product in proteins by Western blotting with streptavidin-HRP. BSA (1 mg/ml), with or without Cu^2+^/PQQ, was incubated in 0.1 M sodium phosphate buffer (pH 7.4) for 72 h at 37 °C. Proteins treated with Cu^2+^/PQQ (1 mM Cu^2+^ plus 1 mM PQQ) were further incubated with 50 mM NaBH_4_ for 1 h on ice. The sample was then treated with 3 mM biotin thioacid in 50 mM Na_2_CO_3_ for 2 h on ice and subjected to native PAGE and Western blotting with streptavidin-HRP. Data are representative of more than five independent experiments. BSA, bovine serum albumin; HRP, horseradish peroxidase; NaBH_4_, sodium borohydride; PQQ, pyrroloquinoline quinine.

To detect 2-piperidinol product in proteins, we utilized an *S*-acylation reaction based on the reaction of *N*-hydroxymethylamines with thioacids (21). In our preliminary experiment, we confirmed that 2-piperidinol (II) indeed produced *S*-acetyl mercapto-2-piperidine upon reaction with thioacetate (data not shown). Then, we tested the reaction of 2-piperidinol (II) with biotin thioacid and detected an intense M + H^+^ at *m*/*z* 570.8, corresponding to the 2-piperidine thioester derivative, by LC–MS (Fig. 8, *A* and *B*). The underlying reaction mechanism of derivatization may include dehydration of 2-piperidinol to a tetrahydropyridine product, followed by the formation of a thioester derivative by the addition of thioacid (Fig. 8*C*). Using this derivatization reaction, we attempted to detect the 2-piperidinol product in proteins by Western blotting with streptavidin-horseradish peroxidase (HRP). Treatment of bovine serum albumin (1 mg/ml) with PQQ in the presence of Cu^2+^ showed a slight expansion of the protein and a shift in mobility on native PAGE analysis (Fig. 8*D*, *left panel*). Western blot analysis of oxidized proteins gave a strong signal because of the binding of biotin thioacid to the protein. In addition, the treatment of the oxidized protein with NaBH_4_ prior to the derivatization with biotin thioacid resulted in partial loss of the signals (Fig. 8*D*, *right panel*).

### Polyphenol-mediated formation of aldehyde–piperidinol products

**Figure 9:**
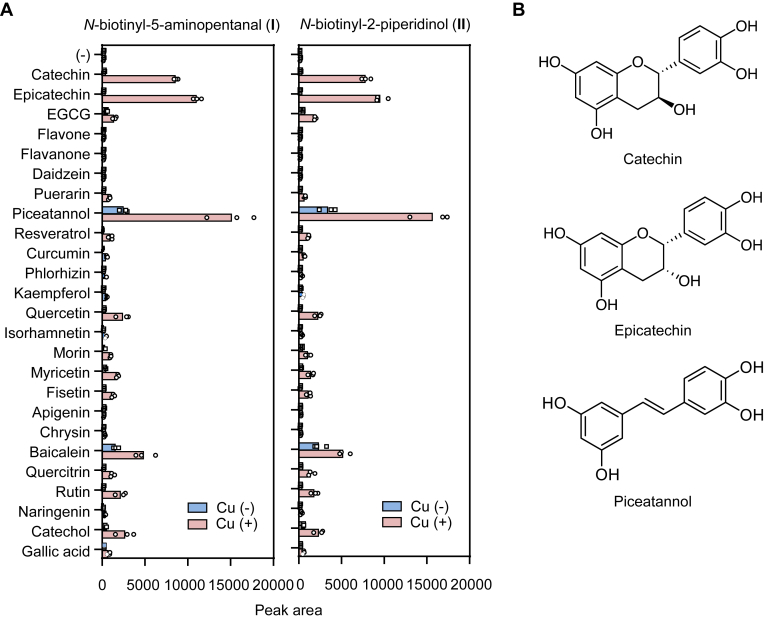
**Oxidation of Bt-APA by polyphenols.***A*, Bt-APA (1 mM) was incubated with 2.5 mM polyphenols in the presence and absence of CuSO_4_ (0.5 mM) in 0.1 M sodium phosphate buffer (pH 7.4) containing 10% DMSO for 24 h at 37 °C. The products comprising aldehyde (I)/piperidinol (II) products were analyzed by LC–ESI–MS/MS in the positive ion mode using multiple reaction monitoring. *B*, chemical structures of catechin, epicatechin, and piceatannol. Bt-APA, *N*-biotinyl-5-aminopentylamine; DMSO, dimethyl sulfoxide.

To obtain insights into the formation of aldehyde–2-piperidinol derivatives by polyphenols, Bt-APA (1 mM) was incubated with polyphenols (2.5 mM) in the presence and absence of copper ions (0.5 mM) in 0.1 M sodium phosphate buffer (pH 7.4) for 24 h, and the products comprising both aldehyde and 2-piperidinol products were analyzed by LC–ESI–MS/MS in the positive ion mode using multiple reaction monitoring. Of interest, not all the polyphenols exhibited an aldehyde/piperidinol–producing activity upon incubation with the lysine analog (Fig. 9*A*). In the absence of copper ions, the potential source of aldehyde–piperidinol products includes piceatannol and baicalein, both of which were much more efficient than EGCG. Copper ions drastically promoted the formation of oxidatively deaminated products. Piceatannol showed the greatest activity, which was followed by catechin, epicatechin, baicalein, quercetin, and catechol. Because these polyphenols contain an *o*-diphenolic structure in common (Fig. 9*B*), it is reasonable to estimate that polyphenol-mediated oxidative deamination may proceed *via* a copper/catechol-driven mechanism. This speculation can be supported by the previous findings that polyphenols, *o*-diphenolic-type polyphenols in particular, gain lysyl oxidase–like activity *via* oxidation to the corresponding *o*-quinone derivatives (10, 13).

### Innate epitope function of aldehyde–piperidinol products

**Figure 10:**
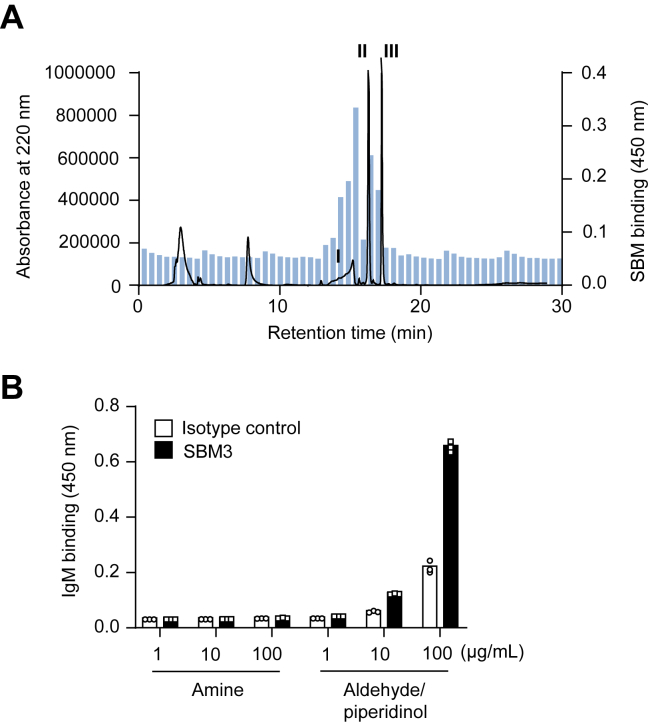
**Innate epitope function of aldehyde–piperidinol products.***A*, ELISA analysis of the HPLC fractions for the immunoreactivity with the IgM mAb SBM3. Bt-APA (3 mM) was incubated with the ESM (10 mg/ml) in 0.1 M sodium phosphate buffer (pH 7.4) for 24 h at 37 °C. The products were monitored at UV 220 nm. *Solid line*, profile of UV absorbance at 220 nm. *Bar*, ELISA analysis. *B*, ELISA analysis of the isolated sample (a mixture of aldehyde–piperidinol). Two IgM mAbs were used: SBM3 raised from the SPF-maintained BALB/c mice, showing the specificity toward the EGCG-modified proteins; and an isotype control IgM (M079-3). Bt-APA, *N*-biotinyl-5-aminopentylamine; EGCG, (-)-epigallocatechin-3-*O*-gallate; ESM, eggshell membrane; IgM, immunoglobulin M; mAb, monoclonal antibody; SPF, specific pathogen-free.

We have shown that polyphenols with lysyl oxidase–like activity can be an endogenous source of innate epitopes recognized by natural IgM antibodies and suggested a mechanism, in which the changes in the electronegative potential of the polyphenol-treated proteins might be involved, at least in part, in the recognition by the antibodies (16). However, the molecular mechanism underlying the formation of the innate epitopes by polyphenols remains unclear. Hence, to determine if oxidation products of Bt-APA function as an innate epitope, we fractionated the products obtained by incubating the lysine analog with ESM by HPLC, and the immunoreactivity of each fraction with natural IgM antibodies was evaluated. For this purpose, IgM mAb SBM3, showing specificity to the EGCG-modified proteins (17), established from specific pathogen-free–maintained BALB/c mice was used. The ELISA analysis of the HPLC fractions indicated that the antibody has an immunoreactivity with multiple fractions containing products I to III (Fig. 10*A*). We then tested the immunoreactivity of the isolated sample (aldehyde–piperidinol mixture) and found that the product was indeed recognized by IgM mAb SBM3 (Fig. 10*B*).

## Discussion

In the present study, to gain more insight into the oxidative deamination of lysine residues, we characterized the oxidation products generated by incubating the lysine analog (Bt-APA) with ESM and established a unique equilibrium between aldehyde and 2-piperidinol products. In addition, based on the presence of such equilibrium, we assessed the lysyl oxidase–like activity of polyphenols by monitoring both products and observed that the reaction was specifically mediated by several *o*-diphenolic-type polyphenols in the presence of copper ions. These data provide the first experimental evidence for the existence of the ring-closed intermediates generated during oxidative deamination of lysine residues. More notably, the aldehyde–2-piperidinol product also functioned as an innate epitope recognized by the natural IgM antibodies. The data provide the basis for the hypothesis that the specificity of the natural IgM for polyphenol-modified proteins may be due, at least in part, to aldehyde–piperidinol products. Furthermore, these findings may indicate that the dynamic equilibrium of oxidatively deaminated lysine between the ring-open and ring-closed intermediates in proteins is associated with the potential beneficial effects of polyphenols.

**Figure 11:**
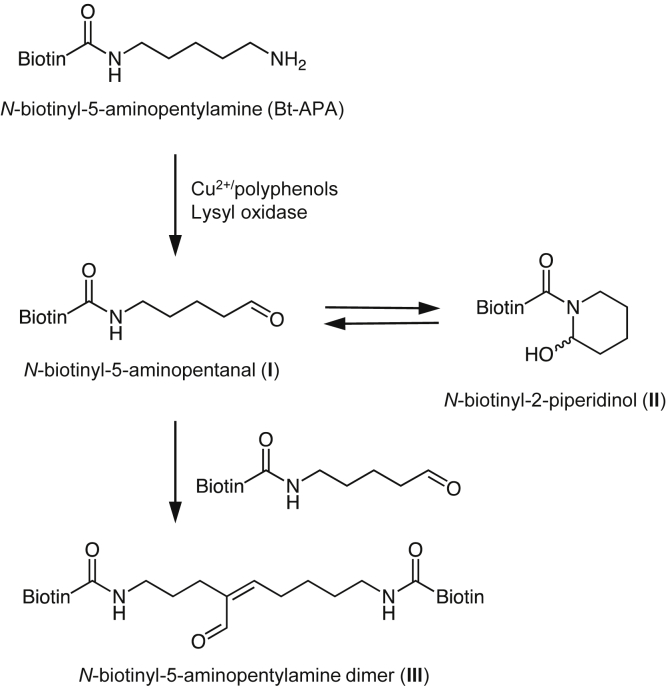
**A proposed mechanism for oxidative deamination of a lysine analog**.

We used Bt-APA as a lysine analog, which provides a common fragment ion at *m/z* 227, throughout this study. It was expected that this characteristic common fragment ion might allow selective analysis of products derived from the lysine analog and facilitate product identification by LC–MS. When Bt-APA was incubated with ESM under physiological conditions, reverse-phase HPLC analysis of the reaction mixture yielded mainly two major products (I and II) and one minor product (III). Incubation of the lysine analog with PQQ in the presence of Cu^2+^ also generated the same products. The data suggest that these products could commonly be generated by the action of polyphenols with lysyl oxidase–like activity. LC–MS data suggested that product I might represent an aldehydic product, *N*-biotinyl-5-aminopentanal, and this hypothesis was confirmed by experiments with NaBH_4_. III was identified as a cross-linking product generated *via* the aldol condensation reaction of two aldehyde species known to be the source of further crosslinks, such as desmosine and its isomer isodesmosine, in elastin and collagen (22). However, no attempt was made to detect these pyridyl crosslinks in this study. Product II was initially predicted to be a tetrahydropyridine product based on MS data but was later identified as a 2-piperidinol product. Figure 11 summarizes the mechanism for the oxidative deamination of Bt-APA. The lysine analog is first converted to the aldehyde (I) and equilibrated with the 2-piperidinol product (II). The aldehyde undergoes a secondary reaction with another aldehyde molecule to form the aldol condensation product (III). As far as we know, this is the first study to show the equilibrium between aldehyde species and ring-closed 2-piperidinol product. At least in terms of the lysyl oxidase–mediated oxidation of Bt-APA, the ring-open structure appears to be more predominant than ring-closed structure, suggesting that equilibrium may favor the formation of crosslinks.

Since the 2-piperidinol product has a chiral carbon center (C-2) and stereogenic nitrogen, it is thought to be composed of two diastereomers because of its equilibrium with the 5-aminopentanal derivative. ^1^H-NMR analysis of the 2-piperidinol product (II) detected two signals at 5.55 and 5.90 ppm corresponding to the methine proton of the hemiaminal group. In addition, two proton signals, 3.60 and 4.06 ppm, corresponding to the methylene group adjacent to the hemiaminal methine group were also detected. The ^13^C-NMR spectrum also showed two signals, 71.92 and 76.50 ppm, corresponding to the methine carbon of hemiaminal. Therefore, in agreement with theory, it was shown that 2-piperidinol products are composed of two configurations. Considering the steric effect of the hydroxy group, it was speculated that the equatorial position is preferable to the axial position of the hydroxyl group. However, the signal intensity of the methine proton in the hemiaminal group revealed that the two stereoisomers might be equally present.

It was previously reported that the formation of 2-piperidinol product was predicted as an intermediate in the process of conversion of aldehyde intermediate to tetrahydropyridine products (20). In the current study, however, no tetrahydropyridine products were detected, and only 2-piperidinol species were detected as cyclic products in the oxidation of lysine analogs with lysyl oxidase and polyphenols. The reason why the tetrahydropyridine product cannot be detected remains unclear, but one plausible explanation for the undetectability of dehydration product is that the rapid interconversion (mutarotation) between ring-open and ring-closed structures is more predominant than the dehydration reaction. In any event, these secondary cyclization reactions may be characteristic of oxidative deamination of lysine residues and are therefore important for understanding the biological impact of oxidized proteins.

By monitoring the aldehyde–piperidinol intermediates, we evaluated the lysyl oxidase–like activity of several polyphenols using ESI–LC–MS/MS and observed that the oxidative deamination was mediated by some polyphenols, including piceatannol, catechin, and epicatechin (Fig. 9). The observation that Cu^2+^ accelerated the formation of oxidation products suggests that the reaction can proceed *via* a metal-catalyzed mechanism. Polyphenols are generally very sensitive to Cu^2+^-catalyzed oxidation. Suzuki *et al.* (23) studied coordination chemistry between PQQ and copper ions and reported the ligation of copper ions to pyridine nitrogen in the carboxyl moiety of PQQ. They also proposed the structures of a copper–PQQ complex and showed that the substrate (amino compound) has access to the *o*-quinone moiety of PQQ, which is activated by the electron-withdrawing effect of copper. Therefore, the formation of such metal complexes coordinated with substrates, and polyphenols may be essential for oxidative deamination by polyphenols.

Natural IgM antibodies play important and diverse roles in health and disease. In particular, its role in invading microbes has been extensively investigated (24, 25). More recent studies highlight their potential role in homeostasis (26), including suppression of inflammation, removal of misfolded proteins, apoptotic and altered cells, and other self-antigens. In addition to these antigens, oxidation-specific epitopes such as oxidized lipoproteins have been shown to constitute important targets for IgM antibodies and innate immunity in general. Based on these protective functions, IgM antibodies are believed to be beneficial in protecting against pathogens and removing cells and molecules that are no longer needed. Moreover, these epitopes can be a signal of an immune response through binding to IgM antibodies (25, 27). Our previous study (16) showed that innate IgM antibodies recognized EGCG-treated proteins and speculated on the involvement of polyphenol-mediated electrical conversion of proteins in antibody recognition. Then, based on the discovery that protein polymerization is involved in the crossreactivity of polyphenol-derived epitopes with IgM, we provided the basis for the hypothesis that they may be due, at least in part, to polymerized proteins (17). However, despite these extensive research efforts on the identification of structural elements of modified proteins involved in recognition by natural antibodies, few studies have clearly shown the structure of the epitope. In this study, taking advantage of the fact that aldehyde–piperidinol products were separable, we investigated the recognition specificity of IgM for products and confirmed that the mAb recognized the product as an innate epitope. This is the first evidence that oxidatively deaminated products have some specific biological effects, such as acting as an innate antigen and eliciting an innate immune response. Meanwhile, our preliminary studies have also shown that polyphenol-mediated product formation does not correlate accurately with antibody recognition of proteins treated with polyphenols (unpublished data). The exact reason for the low correlation remains unknown, but we speculate that it may be due to the multispecificity of the IgM antibody.

Finally, the detection of the aldehyde–piperidinol products in proteins is a critical concern, since 2-piperidinol products are equilibrated with the aldehyde form in aqueous solutions and must be stabilized prior to analysis. Hence, we utilized biotin thioacid as a probe for the selective derivatization of 2-piperidinol product generated on proteins. This method is based on a previous study showing that *N*-hydroxymethylamine, which is in equilibrium with the dehydrated form, can be converted to a thioester derivative by reaction with thioacetate (21). It was expected that the presence of the biotin tag introduced into proteins could allow the 2-piperidinol product to be detected using a series of investigative procedures. Using this probe, Western blotting with streptavidin-HRP detected a strong signal of the oxidized protein (Fig. 8). In addition, the signal was reduced by NaBH_4_ treatment prior to the derivatization with the biotin probe and therefore may be derived from the 2-piperidinol product. Although further studies are needed on the specificity of the method, these data tentatively confirmed the presence of 2-piperidinol in the oxidized protein. The use of LC–MS/MS following derivatization with biotin thioacids may further enable specific detection of 2-piperidinol resulting from oxidative deamination of lysine residues *in vivo*. The development of these methods not only offers structural insights into oxidized proteins but also provides a platform for the development of new biomarkers for human diseases. They also allow us to assess the role of the given pathway in particular pathological conditions.

## Experimental procedures

### Materials

EZ-Link Pentylamine-Biotin (Bt-APA) was obtained from Thermo Fisher Scientific. All the other reagents used in this study were of analytical grade and obtained from commercial sources.

### Lysyl oxidase–mediated oxidation of Bt-APA and *N*^α^-benzoylglycyl-lysine

ESM was used for the lysyl oxidase–mediated reactions (18). Briefly, the ESM isolated from fresh white leghorn hen eggs was thoroughly washed with distilled water and cut into small pieces (5 × 5 mm). Extra moisture of the ESM was removed using filter paper, and then the ESM was weighed. Bt-APA (3 mM) was incubated with 10 mg/ml ESM in 0.1 M sodium phosphate buffer (pH 7.4) for 0 to 48 h at 37 °C. After incubation, the reaction mixtures were analyzed by reverse-phase HPLC on a ChromaNik Sunniest C-18 column (10.0 × 250 mm; ChromaNik) at a flow rate of 4.7 ml/min. A gradient of solvent A (water) with solvent B (acetonitrile) was used as follows: 5% B at 0 to 5 min, 50% B at 20 min, 95% B at 22 min, and 95% B at 25 min. The elution profiles were monitored by absorbance at 220 nm. Each of the separated fractions containing peaks I to III was subjected to LC–ESI–MS analysis using an SQD single quadrupole mass spectrometer (Waters) equipped with the ACQUITY ultraperformance LC (UPLC) system (Waters). The sample injection volumes of 5 μl each were separated on Develosil HB C30-UG (2.0 × 100 mm; Nomura) at the flow rate of 0.3 ml/min. A gradient of solvent A (water) with solvent B (acetonitrile) was used as follows: 5% B at 0 min, 95% B at 8 min, and 95% B at 13 min. An MS scan was performed in the positive ion mode using nitrogen as the nebulizing gas. The experimental conditions were as follows: ion source temperature, 120 °C; desolvation temperature, 350 °C; cone voltage, 10 eV (for *N*-biotinyl-5-aminopentanal [I] and *N*-biotinyl-5-aminopentanal dimer [III]) and 30 eV (for *N*-biotinyl-2-piperidinol [II]); desolvation gas flow rate is 800 l/h.

For the lysyl oxidase–mediated oxidation of *N*^α^-benzoylglycyl-lysine, the peptide (3 mM) was incubated with 10 mg/ml ESM in 0.1 M sodium phosphate buffer (pH 7.4) for 48 h at 37 °C. After incubation, the reaction mixtures were analyzed by LC–ESI–MS analysis using an SQD single quadrupole mass spectrometer (Waters) equipped with an ACQUITY UPLC system (Waters). The sample injection volumes of 5 μl each were separated on Develosil HB C30-UG (2.0 × 100 mm; Nomura) at the flow rate of 0.3 ml/min. A gradient of solvent A (water) with solvent B (acetonitrile) was used as follows: 5% B at 0 min, 95% B at 8 min, and 95% B at 13 min. An MS scan was performed in the positive ion mode using nitrogen as the nebulizing gas. The experimental conditions were as follows: ion source temperature, 120 °C; desolvation temperature, 350 °C; cone voltage, 30 eV; and desolvation gas flow rate, 800 l/h.

### NMR analysis

The reaction mixture was separated by a reverse-phase HPLC as already mentioned. After evaporation, the resulting precipitate was dissolved in heavy water (D_2_O). The ^1^H NMR spectra (500 MHz) and ^13^C NMR spectra (125 MHz) were recorded at 21 °C by a JNM-ECA500 II (JEOL).

The spectra of Bt-APA: ^1^H NMR (500 MHz, D_2_O): δ1.22 to 1.47 (m, 12H), 2.10 (t, *J* = 7.5, 7.5, 2H), 2.64 (d, *J* = 13.5, 1H), 2.72 (t, *J* = 7.0, 7.0, 2H), 2.85 (dd, *J* = 13.0, 5.0, 1H), 3.04 (m, 2H), 3.19 (m, 1H), 4.28 (dd, *J* = 7.5, 4.5, 1H), 4.46 (d, *J* = 8.0, 5.0, 1H). ^13^C NMR (125 MHz, D_2_O): δ23.16, 25.21, 27.70, 27.89, 27.99, 28.01, 35.52, 39.03, 39.72, 55.45, 60.28, 62.13, 165.38, and 176.71. The spectra of a mixture composed of I and II: ^1^H NMR (500 MHz, D_2_O): δ1.24 to 1.76 (m, 10H), 2.11 (t, *J* = 7.0, 7.0, 1H), 2.29 (m, 0.5H), 2.46 (m, 0.6H), 2.65 (d, *J* = 13, 1H), 2.83 (m, 1H), 3.06 (m, 0.9H), 3.19 (m, 1H), 3.60 (m, 0.2H), 4.06 (m, 0.2H), 4.28 (m, 1H), 4.46 (m, 1H), 5.55 (s, 0.2H), 5.90 (s, 0.2H), 9.52 (s, 0.2H). ^13^C NMR (125 MHz, D_2_O): δ17.32, 18.72, 21.48, 24.29, 24.64, 24.89, 25.21, 27.68, 30.03, 30.81, 35.52, 36.61, 36.95, 38.89, 39.15, 39.70, 41.20, 42.74, 55.38, 60.28, 62.10, 71.92, 76.5, 91.01, 165.40, 175.28, 176.71, 208.59. The spectra of III: ^1^H NMR (500 MHz, D_2_O): δ 1.26 to 1.61 (m, 14H), 2.11 (m, 6H), 2.29 (dd, *J* = 14.0, 7.0, 2H), 2.61 (t, *J* = 13.0, 13.0, 2H), 2.82 (m, 2H), 3.01 (m, 2H), 3.06 (m, 2H), 3.16 (m, 2H), 4.25 (m, 2H), 4.44 (m, 2H), 6.67 (t, *J* = 7.0, 7.0, 1H), and 9.12 (s, 1H).

The spectra of a mixture of GK-1 and GK-2: ^1^H NMR (500 MHz, D_2_O): δ1.33 to 2.47 (m, 12H), 4.04 (m, 2H), 4.33 (m, 2H), 5.62 (s, 0.15H), 5.97 (s, 0.15H), 7.43 (t, *J* = 7.0, 7.0, 2H), 7.53 (t, *J* = 7.0, 7.0, 1H), 7.71 (m, 2H), and 9.53 (s, 0.7H). The spectra of GK-3: ^1^H NMR (500 MHz, D_2_O): δ1.28 to 2.57 (m, 12H), 3.93 to 4.04 (m, 6H), 6.56 (m, 1H), 7.38 (m, 4H), 7.48 (m, 2H), 7.67 (m, 4H), 9.03 (s, 1H), 3.16 (m, 2H), 4.25 (m, 2H), 4.44 (m, 2H), 6.67 (t, *J* = 7.0, 7.0, 1H), and 9.12 (s, 1H).

### Reduction by NaBH_4_

The mixture (10 μg/ml) of *N*-biotinyl-5-aminopentanal (I) and *N*-biotinyl-2-piperidinol (II) dissolved in 0.1 M sodium phosphate buffer (pH 7.4) was treated with 10 μl of NaBH_4_ in methanol at the indicated final concentrations for 1 h on ice. Polyphenol-modified protein (1 mg/ml) in 0.1 M sodium phosphate buffer (pH 7.4) was treated with 50 mM NaBH_4_. The reduced product was characterized by LC–ESI–MS, ^1^H NMR, and ^13^C NMR. ^1^H NMR (500 MHz, D_2_O): δ1.19 to 1.58 (m, 12H), 2.10 (t, *J* = 7.0, 7.0, 2H), 2.64 (d, *J* = 13.0, 1H), 2.85 (dd, *J* = 13.0, 2.5, 1H), 3.04 (m, 2H), 3.19 (m, 1H), 3.45 (t, *J* = 6.5, 6.5, 2H), 4.28 (dd, *J* = 8.0, 4.5, 1H), and 4.46 (dd, *J* = 8.0, 5.0, 1H). ^13^C NMR (125 MHz, D_2_O): δ22.45, 25.21, 27.67, 27.84, 28.11, 30.94, 35.53, 39.20, 39.70, 55.40, 60.27, 61.64, 62.10, 165.38, and 176.68.

### Methylation

The mixture (∼1 mg) composed of *N*-biotinyl-5-aminopentanal (I) and *N*-biotinyl-2-piperidinol (II) was dissolved in 1 ml of methanol containing 0.1% TFA. After incubation for 12 h at room temperature, the reaction mixture was evaporated and dissolved in 1 ml of water for the LC–ESI–MS analysis.

### Synthesis of biotin thioacid

Biotin (200 μmol) was dissolved in dimethylformamide. To this solution, diisopropylethylamine (400 μmol) was added. This reaction mixture was then cooled to 4 °C prior to addition of acetic anhydride (200 μmol) and stirred for another 30 min. The reaction mixture was treated with sodium hydrosulfide (220 μmol) and stirred for another 4 h. After drying the reaction mixture, the dried mixture was dissolved in a 3:1 mixture of 0.1% TFA and dimethyl sulfoxide (DMSO) and subjected to a fractionation by HPLC on a ChromaNik Sunniest RP-AQUA column (20.0 × 250 mm; ChromaNik) at a flow rate of 10 ml/min. A gradient of solvent A (water containing 0.1% TFA) with solvent B (acetonitrile containing 0.1% TFA) was used as follows: 20% B at 0 to 5 min, 50% B at 20 min, 95% B at 22 min, and 95% B at 25 min. The elution profiles were monitored by absorbance at 220 nm. Fractions corresponding to the peak of biotin thioacid were dried and stored at −80 °C. The spectra of a mixture of biotin thioacid: ^1^H NMR (500 MHz, DMSO-d_6_): δ1.35 (m, 2H), 1.46 (m, 2H), 1.61 (m, 2H), 2.63 (m, 2H), 2.84 (m, 2H), 3.09 (m, 1H), 4.13 (m, 1H), and 4.30 (m, 1H).

### Biotin labeling of the polyphenol-modified protein

Biotin labeling of the polyphenol-modified protein was performed using the reaction mixtures containing 1.0 mg/ml protein with 3 mM biotin thioacid and 50 mM Na_2_CO_3_. After incubation for 2 h on ice, the protein samples were run on nondenaturing native PAGE. After electrophoresis, the gel was transblotted onto a polyvinylidene difluoride membrane (GE Healthcare), incubated with blocking one (Nacalai Tesque, Inc), washed, and then incubated with streptavidin-HRP (1:5000) for 1 h at room temperature. This procedure was followed by the addition of SuperSignal West Pico PLUS Chemiluminescent Substrate (Thermo Fisher Scientific, Inc). The blots were visualized by ImageQuant LAS4000 (Cytiva).

### Modification of Bt-APA by polyphenols

Bt-APA (1 mM) was incubated with 2.5 mM polyphenols in the presence or absence of 0.5 mM CuSO_4_ in 0.1 M sodium phosphate buffer (pH 7.4) containing 10% DMSO for 24 h at 37 °C. The samples were subjected to an LC–ESI/MS/MS analysis using a TQD triple-stage quadrupole mass spectrometer (Waters) equipped with an ACQUITY UPLC system (Waters). The sample injection volumes of 5 μl each were separated on Develosil HB C30-UG (2.0 × 100 mm; Nomura) at the flow rate of 0.3 ml/min. A gradient of solvent A (water containing 0.1% formic acid) with solvent B (acetonitrile containing 0.1% formic acid) was used as follows: 5% B at 0 min, 95% B at 8 min, and 95% B at 13 min. Multiple reaction monitoring was performed in the positive ion mode using nitrogen as the nebulizing gas. The experimental conditions were as follows: ion source temperature, 120 °C; desolvation temperature, 350 °C; cone voltage, 30 eV; collision energy, 20 eV; desolvation gas flow rate, 800 l/h; cone gas flow rate, 50 l/h; and collision gas, argon. The strategy was designed to detect the product ion (*m/z* 227.2) from the positively ionized *N*-biotinyl-5-aminopentanal (*m/z* 328.2) and *N*-biotinyl-2-piperidinol (*m/z* 328.2) by monitoring the samples transmitting their [M-H_2_O + H]^+^ >227.2 (for *N*-biotinyl-5-aminopentanal) and [M-H_2_O + H]^+^ >227.2 (for *N*-biotinyl-2-piperidinol) transitions. Since *N*-biotinyl-5-aminopentanal and *N*-biotinyl-2-piperidinol were easily dehydrated, we fragmented both dehydrated forms. These two molecules were distinguished by their retention times (*N*-biotinyl-5-aminopentanal: 3.65 min; *N*-biotinyl-2-piperidinol: 4.02 min).

### ELISA

Either the isotype control IgM (1 μg/ml, M079-3, MBL) or monoclonal IgM SBM3 (1 μg/ml) showing specificity toward the EGCG-modified proteins (17) was immobilized onto a 96-well MaxiSorp plate (Nunc) at 4 °C overnight. The plate was washed with PBS containing 0.05% Tween-20 (PBST) and blocked with 200 μl of 2% skim milk in PBST for 60 min at room temperature. After three washes with PBST, a 100-μl aliquot of the solution containing Bt-APA or the mixture of *N*-biotinyl-5-aminopentanal (I) and *N*-biotinyl-2-piperidinol (II) in PBST was added to the wells. After a 1-h incubation, streptavidin-HRP in PBST was added, incubated for 1 h, and then developed using the TMB Ultra substrate (Thermo Scientific). The reaction was terminated by the addition of 2 M sulfuric acid, and the binding to IgM was quantified by measuring the absorbance at 450 nm.

## Data availability

All data are included within this article.

## Conflict of interest

The authors declare that they have no conflicts of interest with the contents of this article.
